# An Open-Type Crossflow Microfluidic Chip for Deformable Droplet Separation Driven by a Centrifugal Field

**DOI:** 10.3390/mi16070774

**Published:** 2025-06-30

**Authors:** Zekun Li, Yongchao Cai, Xiangfu Wei, Cuimin Sun, Wenshen Luo, Hui You

**Affiliations:** 1 School of Mechanical Engineering, Guangxi University, Nanning 530004, China; lizekun1666@163.com (Z.L.); 13381901997@163.com (Y.C.); 2011301035@st.gxu.edu.cn (W.L.); 2 College of Automotive Engineering, Guangxi Transport Vocational and Technical College, Nanning 530023, China; 3 College of Computer and Electronic Information, Guangxi University, 100 East University Road, Nanning 530004, China; cmsun@gxu.edu.cn

**Keywords:** lab-on-a-chip, centrifugal microfluidics, droplet sorting, open crossflow filtration

## Abstract

This study presents an innovative wedge-shaped inlet weir-type microfluidic chip designed to address common issues of clogging and inefficiency in microfiltration processes. Driven solely by centrifugal force, the chip integrates a crossflow separation mechanism and enables selective droplet sorting based on size, without the need for external pumps. Fabricated from PMMA, the device features a central elliptical chamber, a wedge-shaped inlet, and spiral microchannels. These structures leverage shear stress and Dean vortices under centrifugal fields to achieve high-throughput separation of droplets with different diameters. Using water-in-oil emulsions as a model system, we systematically investigated the effects of geometric parameters and rotational speed on separation performance. A theoretical model was developed to derive the critical droplet size based on force balance, accounting for centrifugal force, viscous drag, pressure differentials, and surface tension. Experimental results demonstrate that the chip can effectively separate droplets ranging from 0 to 400 μm in diameter at 200 rpm, achieving a sorting efficiency of up to 72% and a separation threshold (cutoff accuracy) of 98.2%. Fluorescence analysis confirmed the absence of cross-contamination during single-chip operation. This work offers a structure-guided, efficient, and contamination-free droplet sorting strategy with broad potential applications in biomedical diagnostics and drug screening.

## 1. Introduction

Microfluidic chips, also known as lab-on-a-chip (LOC) or micro total analysis systems (μTAS) [1], have rapidly advanced in recent years with the development of ultra-precision manufacturing technologies. They have emerged as essential platforms for microscale biochemical operations. These systems integrate multiple functions—such as sample preparation, mixing, reaction, separation, and detection—on a single chip [2]. Featuring miniaturization, high throughput, low power consumption, and fast response, microfluidic technologies are widely applied in biomedical diagnostics, environmental monitoring, drug screening, and chemical synthesis [3].

Among various microfluidic approaches, droplet-based microfluidics has gained significant attention [4]. It enables the generation and manipulation of droplets in immiscible multiphase flows within microchannels [5], offering advantages such as operational flexibility, minimal reagent consumption, and cost efficiency [6]. High-throughput droplet sorting and purification are central to droplet microfluidic systems and are critical for the accurate processing of complex samples [7]. However, existing sorting techniques still face challenges in terms of efficiency, selectivity, and system complexity.

Droplet sorting methods are generally categorized into active and passive approaches. Active methods utilize external fields—such as acoustic [8], magnetic [9], pneumatic [10], or electric fields [11]—to achieve precise control. While highly accurate, these systems often involve bulky and complex setups, making it difficult to balance throughput with biocompatibility. In contrast, passive sorting relies on the hydrodynamic interactions between droplets and microstructures. These systems are structurally simple, easy to integrate, and well suited for size-based sorting. Representative techniques include microfiltration [12], pinched flow fractionation [13], inertial sorting [14], and deterministic lateral displacement (DLD) [15].

Among these, microfiltration stands out as a size-exclusion-based passive sorting strategy with strong adaptability to droplet applications. Common structural designs include membranes, pillar arrays, and weirs [16]. Microfiltration is especially efficient when processing large sample volumes. However, during continuous operation, microchannel clogging frequently occurs, significantly compromising flow stability and sorting accuracy. Various strategies have been proposed to address this issue, including oscillatory flow [17], mechanical vibration [18], and crossflow filtration [19]. For instance, Shevkoplyas et al. [20] and Seki et al. [21] demonstrated enhanced precision and throughput by incorporating controlled incremental fluid removal strategies in pillar-based crossflow systems. Nonetheless, mechanical vibration may adversely affect biological activity, and crossflow mechanisms struggle to remove non-specifically adsorbed droplets or particles. Additionally, oscillatory flows may lead to secondary clogging during repeated filtration cycles. Thus, clogging remains a critical bottleneck limiting the practical application of microfiltration technologies.

To address this challenge, we propose a centrifugal microfluidic chip integrating a wedge-shaped inlet and a weir-based structure—a variant of crossflow filtration. The chip employs centrifugal force to actively deflect and sort droplets, while the wedge structure induces a shear gradient to enhance size-based separation accuracy. The sorting threshold is primarily determined by the microstructure, thereby reducing dependence on dynamic control. High-speed centrifugation enables the active removal of residual samples and clogging particles, forming a self-cleaning operational loop. This design significantly alleviates clogging, enhances reusability and stability, and offers a new pathway for high-throughput, multi-sample processing in microfluidic systems.

## 2. Materials and Methods

### 2.1. Experimental Setup and System Configuration

**Figure 1:**
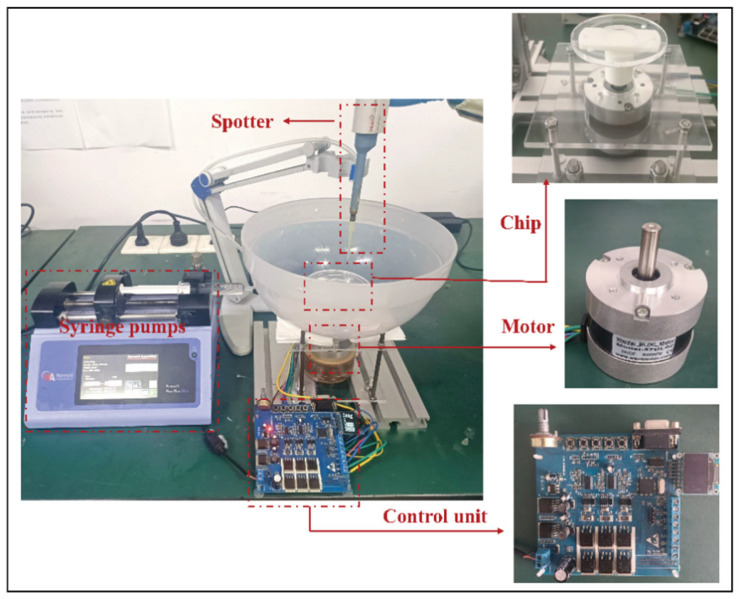
Schematic diagram of the experimental setup components.

As illustrated in Figure 1, the centrifugal microfiltration and droplet sorting system comprises five main modules:

Separation module: This is the core of the system, consisting of a microfluidic sorting chip and a sample collection unit. The chip performs droplet screening, and the sorted samples are directed into designated collection chambers.

Sample injection module: An automated dispenser introduces the emulsion samples into the central inlet of the rotating chip, initiating the sorting process.

Sample elution module: After separation, a precision oil pump (ELITE PICOSINGLE, Harvard Apparatus, Holliston, MA, USA) injects the oil phase into the chip’s central inlet to flush out any residual droplets from the channels. During this step, the injection flow rate must be carefully controlled to match the chip’s processing throughput, maintaining a stable hydrostatic pressure and preventing pressure fluctuations that could affect separation accuracy.

Driving module: A programmable motor: Wantai BLDC Motor (Jiangsu Wantai Motor Co., Ltd., Yancheng, China) (operational speed range: 0–1000 ± 5 rpm) and its control unit generate the required centrifugal field by precisely rotating the chip.

Imaging and analysis module: Following separation, emulsions collected from each chamber are transferred to microscope slides using a pipette. Images are captured using a handheld microscope: Dino-Lite Digital Microscope (VIDY Optical (Wuxi) Co., Ltd., Wuxi, China) and subsequently analyzed using Premiere Pro 2021 and ImageJ software, version 1.54p for image processing and quantitative assessment.

### 2.2. Design and Fabrication of the Microfluidic Chip

The microfiltration chip developed in this study consists of two polymethyl methacrylate (PMMA) substrates, each 3 mm thick, assembled using precision micromachining and chemical bonding techniques. The top layer incorporates all functional microstructures, including an elliptical chamber, a wedge-shaped inlet, and spiral outlet channels, while the bottom layer is a flat, unstructured plate serving as a sealing substrate.

At the core of the chip is a large elliptical chamber, which functions as the primary zone for droplet separation. The chamber has a semi-major axis of a = 23 mm, a semi-minor axis of b = 18 mm, and a depth of 150 μm. Microchannels (0.5 mm wide, 150 μm deep) are connected to both ends of the chamber to guide and collect separated droplets. A compound inlet structure is located at the center of the chamber, comprising a circular injection port and a recessed wedge-shaped opening. The circular port is directly connected to the chamber, while the wedge forms a sloped transition zone on one side. Under centrifugal force, this structure induces flow asymmetry, promoting crossflow separation along the wedge surface.

**Figure 2:**
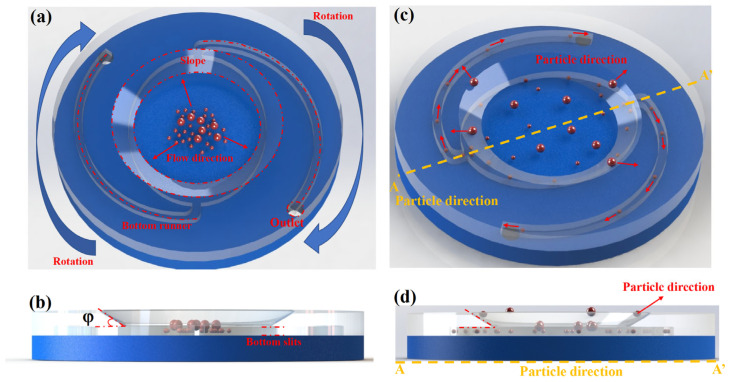
Schematic illustrations of sample processing and sorting mechanism in the microfiltration chip: (**a**) Sample injection mechanism. (**b**) Cross-sectional view of the inlet region. (**c**) Diagram of droplet separation process. (**d**) Cross-sectional view of separation behavior.

The angle φ formed between the wedge-shaped outlet and the horizontal plane of the chip (termed the separation angle) governs the coupling between tangential flow and normal resistance, thereby modulating the trajectory of incoming droplets and significantly influencing separation sensitivity (see Figure 2b).

During continuous-flow operation (Figure 2c), samples are introduced at the center of the chip. Centrifugal acceleration and Coriolis forces together generate a stable laminar flow field along the major axis of the elliptical chamber. The local shear stress induced by the wedge-shaped inlet, combined with geometric confinement from the narrow gap, causes droplets of different sizes to exhibit distinct migration behaviors. Small-sized water-in-oil droplets, upon entering the gap of height δ = 150 μm, follow the curvature of the chamber and are directed laterally by Dean vortices, eventually being collected into the two spiral outlet channels (channel width w = 0.5 mm). In contrast, larger droplets undergo lateral displacement due to inertial and shear effects at the wedge interface (φ = 30°), sliding along the sloped surface and being ejected from the main flow path—effectively realizing a “throwing-off” mode of separation.

This asymmetric shear-based crossflow design enables efficient, size-selective exclusion of deformable droplets. Prior to sample injection, the microchannels must be fully pre-filled with the continuous phase to eliminate capillary-driven wetting effects and prevent bubble formation, which could interfere with the separation process. After separation, custom pipette tips are used to transfer the collected samples from the side channels into external collection reservoirs for downstream analysis.

The entire sorting process operates without external pumps, relying solely on structural design and centrifugal force, demonstrating high structural dependency and efficient passive droplet sorting.

### 2.3. Sample Preparation

To evaluate the droplet sorting performance of the designed microfiltration chip, a water-in-oil (W/O) emulsion system was selected as the model sample. The emulsion consisted of #5 mineral oil (continuous phase) and deionized water (Dispersed phase, product code: LWFS31101T, Cascada, Irvine, CA, USA)), mimicking typical microreactor environments widely used in biochemical assays and droplet-based platforms [22].

To enhance imaging resolution and facilitate visual tracking under a microscope, 4% *v*/*v* of food-grade Carmine Red dye (model: TSBR-W04A, Tianxu Biotech, Shanghai, China was added to the dispersed phase. Additionally, 3% *v*/*v* of the nonionic surfactant ABIL EM90 (Evonik, Essen, Germany) was introduced into the continuous phase to improve emulsion stability and prevent droplet coalescence.

All experiments were conducted under controlled ambient conditions (25 ± 0.5 °C) to ensure consistency and reliability of the emulsion’s physicochemical properties. The key parameters of the modified phases, including density, viscosity, and interfacial tension, are summarized in Table 1.

**Figure 3:**
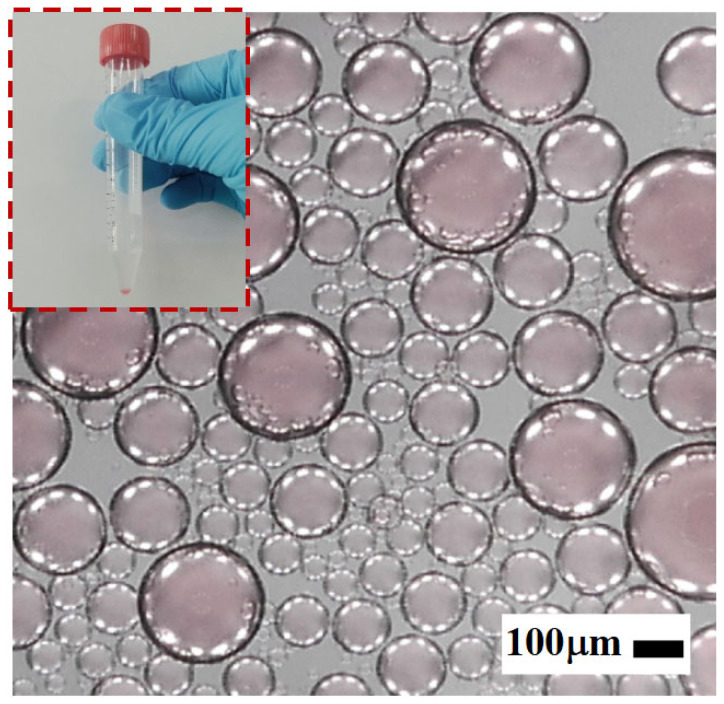
Preparation of water-in-oil microdroplets via mechanical shaking.

As illustrated in Figure 3, the emulsion used in the experiments was prepared via mechanical emulsification. Specifically, a predetermined volume of the aqueous phase was slowly added dropwise into a centrifuge tube preloaded with the continuous phase (mineral oil mixed with surfactant). The mixture was then vigorously agitated using a mechanical shaker to ensure thorough emulsification. The shaking parameters—frequency and duration—were optimized to generate emulsions with a broad droplet size distribution.

The resulting water-in-oil droplets exhibited diameters primarily in the range of 0–400 μm, making them well suited for evaluating the structural selectivity and sorting performance of the proposed microfiltration chip.

## 3. Results and Discussion

### 3.1. Theoretical Derivation of the Critical Droplet Sorting Diameter

**Figure 4:**
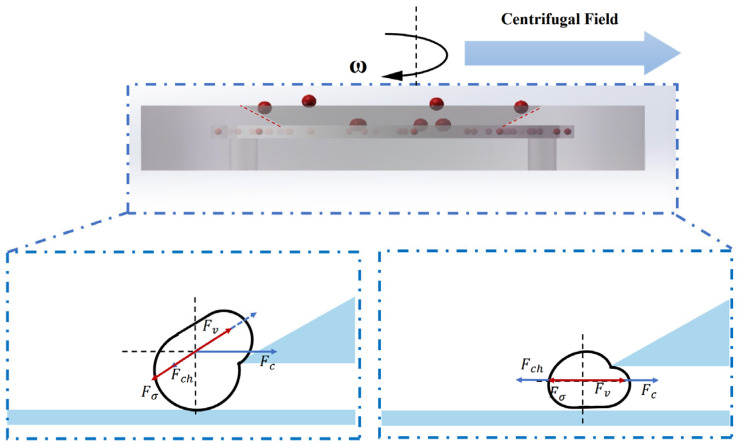
Schematic of droplet dynamics and force interactions within the microfiltration chip under centrifugal field.

Figure 4 illustrates the motion and force interactions of microdroplets within the microfiltration chip under centrifugal actuation. The droplet behavior in the sorting region is governed by a combination of hydrodynamic forces, including centrifugal force, viscous drag, pressure difference across the slit region, and interfacial tension. These forces collectively determine whether a droplet can migrate through the slit into the collection channel (for smaller droplets) or be laterally displaced along the sloped surface and rejected (for larger droplets).

This section systematically analyzes the dominant forces acting on the droplets and establishes a theoretical model to calculate the critical sorting diameter. The model provides a quantitative framework to interpret experimental results and to guide structural optimization of the microfiltration chip.

(1) During the rotation of the chip, a droplet is subjected to a centrifugal force that drives it radially outward. The magnitude of this force is given by

(1) $$F_{c}=\frac{\pi }{6}D_{d}^{3}\left(\rho_{d}-\rho_{f}\right)r\omega^{2}$$

where $D_{d}$ is the droplet diameter; $\rho_{d}$ and $\rho_{f}$ denote the densities of the dispersed phase (water) and continuous phase (oil), respectively; *r* is the radial distance of the droplet from the rotation center of the chip; and ω is the angular velocity.

When $D_{d}$ > $D_{dc}$, the droplet size is relatively large, and the centrifugal force dominates its motion, causing the droplet to be “flung out” along the inclined ramp.

(2) The viscous drag force exerted on the droplet by the continuous phase flow is given by

(2) $$F_{v}=3\pi \mu_{f}D_{d}(U_{f}^{*}-U_{d})$$

where $U_{f}^{*}$ denotes the centerline velocity of the channel flow [23].

(3) $$U_{f}^{*}=\frac{Q}{A}\cdot \left(1-{\left(\frac{y}{h}\right)}^{2}\right)$$

For smaller droplets, the viscous drag facilitates their lateral displacement into the gap.

(3) The driving force exerted on the droplet due to the pressure difference across the gap is expressed as

(4) $$F_{ch}=\Delta P_{ch}\cdot A_{eff}$$

The effective acting area $A_{eff}$ is given by

(5) $$A_{eff}=\frac{\pi D_{d}^{2}}{4}$$

(4) The resistance caused by interfacial tension when the droplet deforms to pass through the gap is expressed as [17]

(6) $$F_{\sigma }=\sigma \cdot \pi D_{d}\left(\frac{1}{R_{1}}-\frac{1}{R_{2}}\right)$$

Considering the stable mass and flow velocity of the microdroplets, their inertial effects are neglected.

(5) Dean Flow Perturbation. Due to radial shear and curvature in the microchannel, droplets are also influenced by secondary transverse flow (Dean flow), characterized by the Dean number:

(7) $$De=\frac{Re\cdot \sqrt{h/2R_{c}}}{2}$$

The Reynolds number within the gap is defined as

(8) $$Re=\frac{\rho_{f}U_{f}h}{\mu_{f}}$$

The resulting Dean velocity perturbation is approximated by [24]

(9) $$U_{Dean}\approx \alpha \cdot U_{f}\cdot De^{1.63}$$

which generates an additional lateral drag force on the droplet:

(10) $$F_{Dean}=3\pi \mu_{f}D_{d}U_{Dean}$$

Dean flow enhances the lateral migration of small droplets toward the gap and significantly affects the initial droplet position distribution.

Critical Mechanical Equilibrium Criterion: When a droplet just passes through the gap, the forces acting on it reach a balance:

(11) $$F_{c}\cos\theta +F_{D}+F_{Dean}=F_{ch}+F_{\sigma }$$

By simplification and grouping of relevant terms, an approximate expression for the critical droplet diameter is obtained:

(12) $$D_{dc}\approx \sqrt[{3}]{\frac{3\mu_{f}h^{2}\tan\theta \rho_{f}\left(1+\beta D_{e}^{2}\right)}{2\left(\rho_{d}-\rho_{f}\right)\omega^{2}}}$$

In summary, the droplet sorting capability of the microfiltration chip is fundamentally governed by the dynamic balance among centrifugal force, viscous drag, pressure difference, and interfacial tension. By adjusting structural parameters such as gap depth and slope angle, the chip can switch between “high-precision” and “high-throughput” sorting modes. After the sorting process, increasing the rotational speed to 600 rpm further enhances the centrifugal force, effectively clearing residual materials and preventing clogging.

### 3.2. Effect of Wedge Angle φ on Emulsion Separation

This study systematically investigates the regulatory effect of the wedge structure in the microfiltration chip on the separation behavior of heterogeneous water-in-oil emulsions. To characterize the emulsion composition, the dispersed phase volume fraction $A_{c}$ is introduced and defined as follows:

(13) $$A_{c}=\nu_{c}/\left(\nu_{c}+\nu_{b}\right)\times 100\%$$

where $\nu_{c}$ and $\nu_{b}$ represent the volumes of the dispersed and continuous phases, respectively.

**Figure 5:**
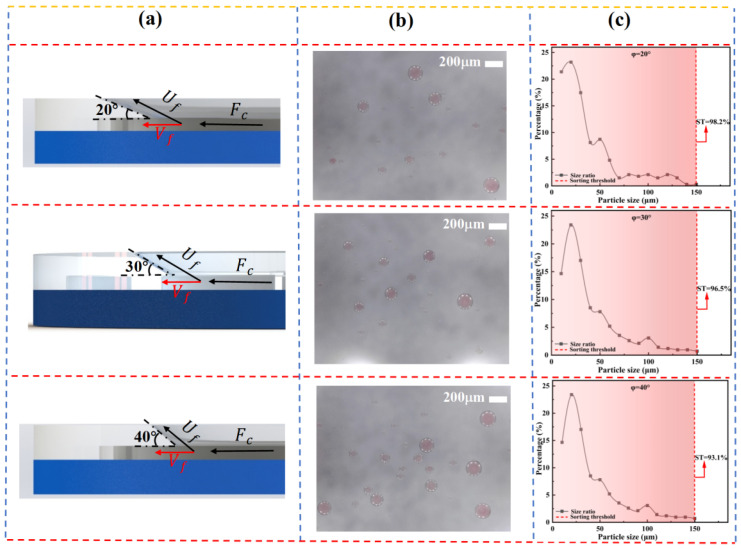
Effect of wedge angle on droplet separation performance at a slit depth of 100 μm: (**a**) Schematic of wedge angles. (**b**) Optical images of droplet separation outcomes. (**c**) Droplet size distribution and variation of separation threshold.

To evaluate the influence of wedge angle, three microfluidic chips with wedge angles of ϕ = 20°, 30°, and 40 were employed. A water-in-oil emulsion with a dispersed phase volume fraction of 12.2% *v*/*v* was introduced into the chip inlet (see Figure 5a). Separation experiments were conducted at a centrifugal speed of ω = 100 rpm, with each condition repeated independently more than 10 times to minimize systematic and random errors.

Under the centrifugal field, droplets with smaller diameters experienced a net force directed toward the gap and were able to pass through the slit into the elliptical chamber. Inside the chamber, these droplets underwent radial migration, driven by a combination of Dean vortices and Coriolis forces. Gradually, the small droplets focused along the chamber sidewalls and were guided into the spiral outlet channels, allowing for efficient collection.

In contrast, larger droplets, unable to pass through the gap, were diverted from the main flow. These droplets were subsequently deflected along the wedge slope by their inertia and effectively ejected from the flow path, thereby achieving size-selective separation.

As shown in Figure 5, the spatial distribution of droplets under different wedge angles φ is presented. The results indicate that the total number of collected droplets increases significantly with increasing φ. This can be attributed to the rise in the critical separation diameter, meaning that larger wedge angles facilitate the passage of smaller droplets through the slit, while making it more difficult for larger droplets to migrate along the sloped surface. In addition, Figure 5c demonstrates a gradual increase in the average droplet size with increasing φ, suggesting that the wedge angle has a regulatory effect on the size range of the separated droplets.

To quantitatively evaluate the chip’s capability for selective capture of target droplets, two key performance indicators are introduced: Collection Efficiency (*CE*) and Separation Limit (*SL*). The Collection Efficiency (*CE*) is defined as the percentage of droplets within the target size range that are successfully collected by the chip relative to the total number of droplets of that size:

(14) $$CE=\frac{N_{target}}{N_{total}}\times 100\%$$

The Separation Limit (*SL*) is defined as the proportion of target-sized droplets among all collected droplets, which serves to evaluate the degree of contamination by non-target-sized droplets:

(15) $$SL=\frac{N_{target}}{N_{collected}}\times 100\%$$

**Figure 6:**
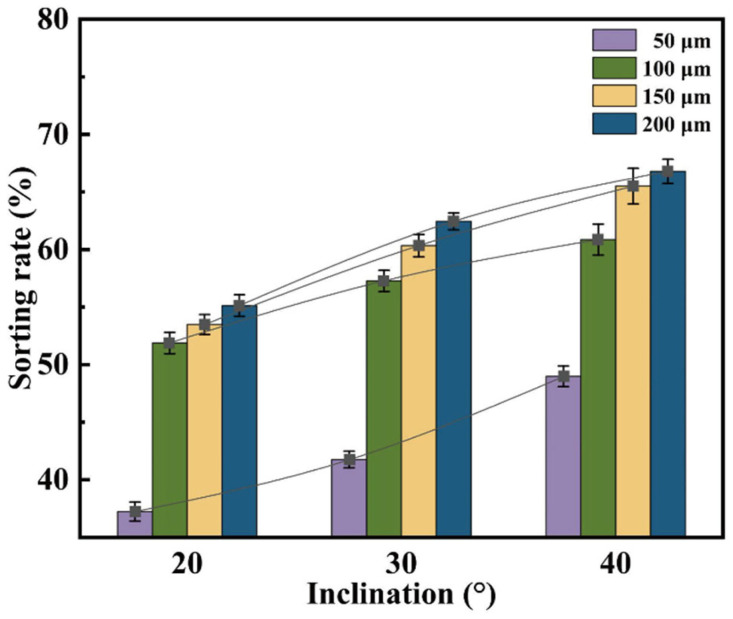
Effect of wedge angle on the separation efficiency of the microfiltration chip.

Figure 6 illustrates the effect of wedge angle on separation efficiency at different gap depths (h = 50, 100, 150, and 200 μm). It can be observed that, regardless of the gap depth, the Collection Efficiency (*CE*) increases linearly with increasing φ. This trend indicates that the shear stress gradient induced by the sloped guiding structure is the primary driving force for enhancing separation performance. Notably, when h = 200 μm, a wedge angle of φ = 40° yields a CE of 67.2%, demonstrating excellent size selectivity.

**Figure 7:**
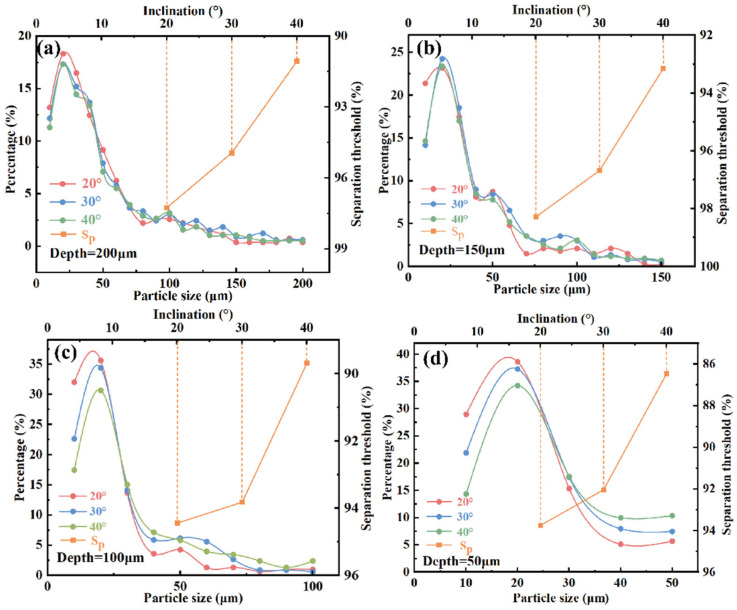
Effect of slit depth and slope angle on the droplet size sorting of oil-in-water emulsions: (**a**) slit depth of 200 μm; (**b**) slit depth of 150 μm; (**c**) slit depth of 100 μm; (**d**) slit depth of 50 μm.

Further analysis of Figure 7 reveals significant differences in the response of droplet size distribution to varying φ at different h values. When h = 200 μm, the size distributions under different φ conditions converge, indicating that at this scale, the influence of the slope angle on sorting behavior is diminished. In contrast, as h decreases to 100 μm or below, the size distribution exhibits clear differentiation with respect to φ. Notably, at h = 50 μm, the sorting performance is highly sensitive to φ, confirming the coupling effect between microstructure geometry and droplet size.

From the perspective of sorting limit (*SL*), analysis of slope angle shows that at a smaller angle (20°), droplets more readily slide along the slope into the slit, resulting in a smoother sorting process. This effect is particularly pronounced at slit depths of 150 μm and 200 μm, where the sorting limits reach optimal values of 98.2% and 97.2%, respectively. Although a slope angle of ϕ = 40° significantly improves sorting efficiency, it also raises the risk of non-target droplets being entrained, thereby reducing sorting specificity. Therefore, optimization of microfilter chip structural parameters must balance sorting efficiency and selectivity to achieve high-precision, high-purity separation.

In summary, slit depth and slope angle exhibit a significant synergistic effect during sorting. When designing microfluidic chips, both parameters should be considered jointly to optimize the balance between sorting limit and sorting efficiency for target droplet size selection and throughput.

### 3.3. Effect of Slit Depth hhh on Emulsion Separation Performance

Compared with other droplet sorting techniques, the microfilter chip designed in this study offers a significant advantage: it can directly process high-concentration emulsions without requiring pre-dilution, thereby greatly enhancing sorting efficiency and facilitating subsequent enrichment.

**Figure 8:**
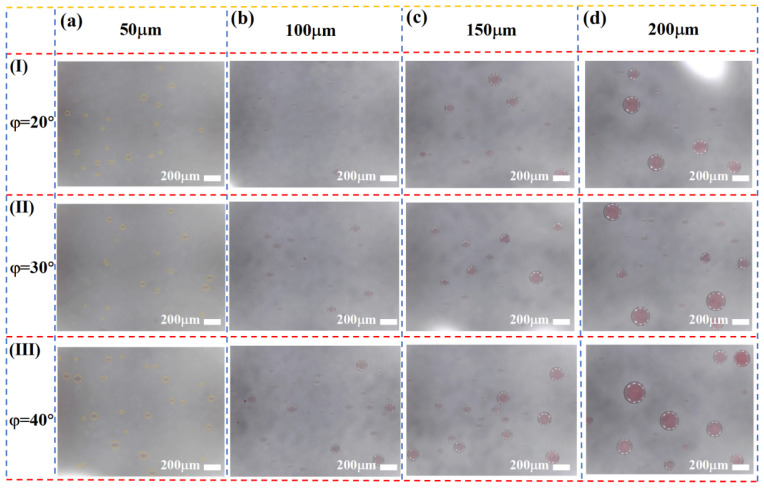
Photographs showing the size distribution of oil-in-water emulsion droplets sorted by varying slit depths.

Figure 8 illustrates the actual size distribution of oil-in-water emulsions collected by the chip under varying slit depths h. As shown, the average droplet size of the collected emulsion increases significantly with increasing slit depth, indicating a clear correlation between droplet size and slit geometry. A deeper slit allows a larger critical droplet diameter to pass through, enabling more large droplets to be effectively collected. Based on the critical sorting size model established herein, increasing slit depth reduces the geometric resistance encountered by large droplets when passing through the slit, thus enhancing their penetration probability. Consequently, a substantial collection of large droplets is observed at larger hhh values, consistent with theoretical predictions. This result further validates the effectiveness and tunability of the wedge-shaped weir microstructure in controlling size-selective droplet sorting.

**Figure 9:**
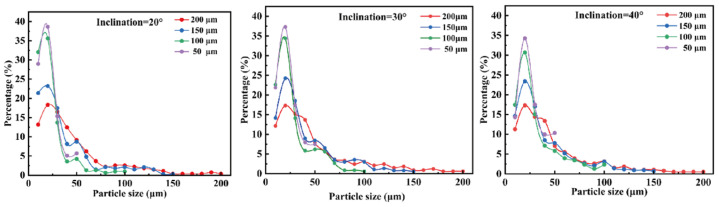
Influence of slit depth on the droplet size distribution of oil-in-water emulsions.

To systematically evaluate the impact of slit depth on sorting performance, statistical analyses were conducted on the quantity and size distribution of collected droplets. Figure 9 presents the proportional distribution of droplets across different size ranges under varying slit depth conditions.

The results indicate that when the slit depth is small, small droplets dominate the collected population, demonstrating a pronounced screening effect. Shallower slits impose stronger geometric constraints that inhibit the passage of large droplets, thereby enhancing size selectivity and sorting purity. Conversely, as the slit depth increases, the passage rate of large droplets significantly rises, especially at larger slope angles. This is because the increased slit depth enlarges the available space for droplets to pass through, reducing the exclusion of large droplets and thus affecting sorting accuracy and selectivity. Additionally, droplet motion within the channel is influenced by the coupling of slit depth and slope angle: shallow slits intensify confinement and fluid resistance, making droplets more readily guided by the slope into the sorting path; deeper slits reduce flow restrictions, allowing some large droplets to bypass the slope guidance and enter the slit region, weakening size-based sorting specificity.

In summary, precise design of slit depth enables efficient and tunable droplet screening and separation under varying emulsion concentrations and droplet size conditions.

### 3.4. Combined Effect of Ramp Angle and Gap Depth on Droplet Separation

**Figure 10:**
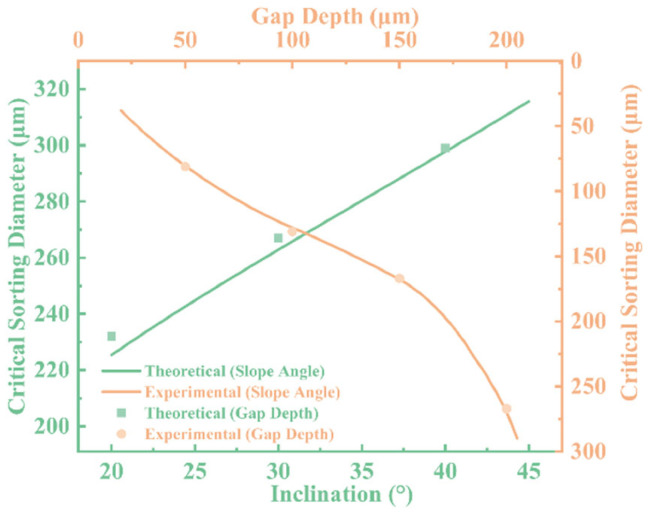
Comparison of theoretical and experimental critical droplet diameters under varying ramp angles and gap depths.

To systematically evaluate the influence of microfiltration chip geometry on droplet sorting behavior, we established a theoretical model to predict the critical separation diameter and validated it experimentally under varying ramp angles (φ) and gap depths (h). As shown in Figure 10, we investigated the effect of ramp angle at a fixed gap depth of 200 μm, and the influence of gap depth at a fixed ramp angle of 30°. A comparison between the theoretical predictions and experimental data reveals good agreement in the overall trends, confirming the validity and applicability of the proposed model.

The results indicate that the critical separation diameter increases with increasing ramp angle. This trend suggests that steeper ramps restrict lateral inertial deviation of droplets, allowing larger droplets to enter the gap more readily and thereby reducing separation selectivity and precision. On the other hand, gap depth also has a significant impact on the critical diameter. As the gap increases from 100 μm to 150 μm and 200 μm, the critical diameter correspondingly increases, indicating that a larger gap provides a broader passage for droplets, weakening the structural constraints and increasing the probability of passage.

Overall, the consistency between the theoretical and experimental results demonstrates the model’s predictive capability under various structural conditions. Furthermore, it provides a quantitative foundation for the structural optimization of microfiltration chips. By tuning both ramp angle and gap depth in tandem, efficient and controllable sorting within the target droplet size range can be achieved, offering theoretical support and design reference for functional microfluidic platforms.

**Figure 11:**
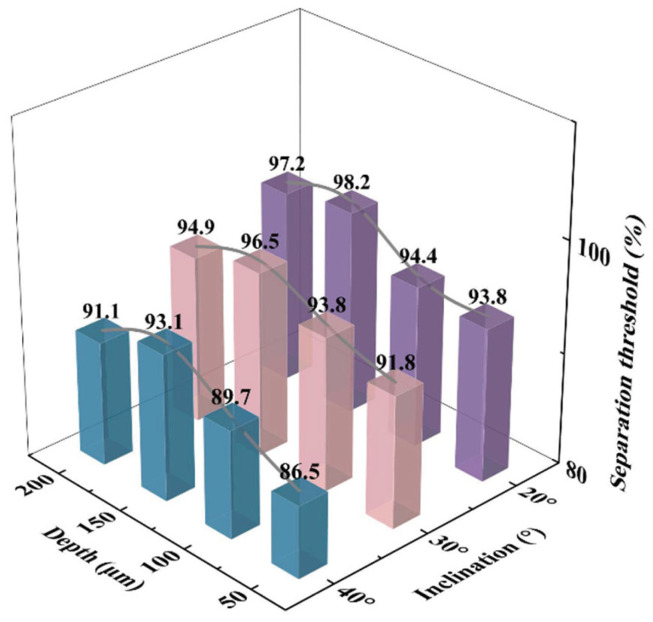
Synergistic regulation of sorting threshold in microfiltration chips by gap depth and ramp angle.

As shown in Figure 11, the sorting selectivity, characterized by the sorting threshold (defined as the proportion of target-size droplets among the collected droplets), was evaluated under different combinations of gap depth and ramp angle.

When the gap depth was 50 μm, the sorting threshold was notably lower than that at larger depths (100 μm, 150 μm, and 200 μm), suggesting that narrow gaps impede droplet passage, particularly for larger droplets. In this case, larger droplets tend to be expelled at the ramp, while smaller ones are more likely to be collected, leading to reduced selectivity. Under the combination of φ = 40° and h = 50 μm, the sorting threshold was the lowest at 86.5%, indicating the highest impurity capture rate. As the gap depth increased, droplet permeability improved significantly, leading to higher sorting thresholds. For gap depths of 100 μm, 150 μm, and 200 μm, the sorting thresholds reached 89.7%, 93.1%, and 91.1%, respectively. However, when the gap became too large (e.g., 200 μm), oversized droplets beyond the target range also passed through, slightly compromising the sorting precision.

With respect to ramp angle, the highest overall sorting performance was observed at φ = 20°, where the sorting thresholds reached 98.2% and 97.2% under gap depths of 150 μm and 200 μm, respectively. Shallower ramps promote smooth sliding of droplets along the surface, enabling effective lateral displacement and enhancing both stability and selectivity. At φ = 30°, the sorting threshold showed a slight decline (e.g., 93.8% at h = 150 μm) but still maintained good performance, possibly due to enhanced inertial effects allowing some impurity droplets to enter the gap. A significant decrease in sorting threshold was observed at φ = 40°, particularly at shallower gap depths (50 μm and 100 μm), where increased ramp steepness introduced stronger droplet perturbation and lateral deviation, elevating the risk of non-target droplet collection and reducing overall performance.

These results highlight a clear coupling effect between ramp angle and gap depth. Smaller gaps limit droplet permeability, while steeper ramp angles exacerbate droplet trajectory instability, ultimately undermining selective sorting. Therefore, optimal chip design should consider the interplay between these two parameters to balance sorting efficiency and selectivity. Our study identifies the combination of a 200 μm gap depth and a 30° ramp angle as achieving an effective compromise, enabling both high sorting efficiency and selectivity across a broader target size range.

### 3.5. Effect of Rotation Speed on Microfilter Chip Sorting Performance

The rotation speed of the disk primarily influences sorting performance by affecting droplet velocity, centrifugal force magnitude, and the resulting hydrodynamic behavior. In this study, rotation speed serves as a key control parameter, significantly regulating droplet migration paths within the chip and their final sorting outcomes.

**Figure 12:**
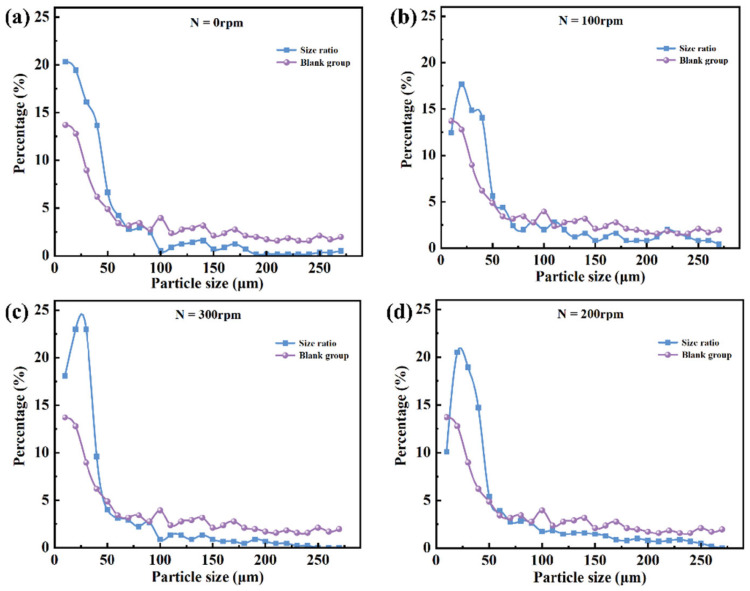
Comparison of droplet size distributions before and after sorting under different rotation speed conditions: (**a**) Size distribution of droplets under 0 rpm, dominated by capillary forces. (**b**) Size distribution at 100 rpm rotational speed. (**c**) Size distribution at 200 rpm rotational speed. (**d**) Size distribution at 300 rpm rotational speed.

Given that the chip exhibits relatively balanced sorting performance at a slit depth h = 200 μm and slope angle of 30°, the comparison between the droplet size distributions before and after sorting under various rotation speeds is presented in Figure 12. As rotation speed increases, the proportion of large droplets in the sorted population decreases markedly, indicating that the enhanced centrifugal force at higher speeds effectively promotes the migration and passage of smaller droplets through the slit. Meanwhile, larger droplets are more readily “thrown off” by the slope due to their greater inertia, reducing their likelihood of mistakenly entering the collection region. This significantly improves size selectivity and sorting accuracy. Experimental results demonstrate that appropriate adjustment of rotation speed enables precise sorting of droplets with varying sizes, highlighting the system’s adaptability and tunable parameters.

**Figure 13:**
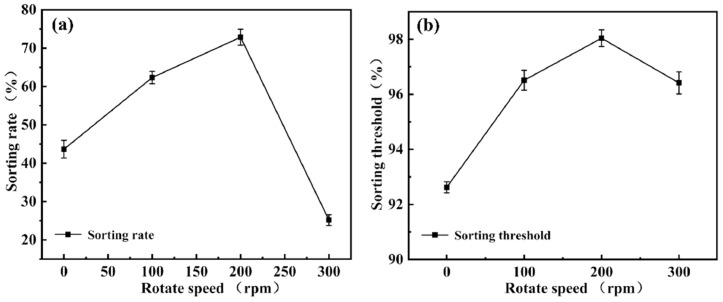
Sorting efficiency and sorting limit of the chip at different rotation speeds: (**a**) Sorting efficiency of the chip at different rotational speeds. (**b**) Sorting threshold of the chip at different rotational speeds.

Figure 13 further illustrates the combined effects of rotation speed variation on droplet sorting behavior and sorting limit. Overall, sorting performance exhibits a “rise-then-fall” trend with increasing rotation speed, indicating the presence of an optimal speed range.

At 0 rpm, droplet movement relies primarily on capillary forces. Due to the low flow velocity and dominant surface tension effects, droplets move slowly and unstably within the microchannels, leading to clogging or erroneous collection of large droplets, and resulting in low sorting efficiency and accuracy. As the rotation speed increases to 100 rpm, centrifugal force begins to influence droplet behavior. Large droplets deviate from the main flow path due to inertia, reducing miscollection, but the relatively low flow velocity limits improvement in sorting smaller droplets.

When the speed further increases to 200 rpm, droplets acquire sufficient kinetic energy; small droplets stably pass through the slit, while large droplets are effectively deflected by inertia, yielding optimal sorting performance. Experimental data show that at this speed, the sorting efficiency reaches approximately 72%, and the sorting limit approaches 98%, representing the system’s optimal operating condition. Under these conditions, sorting demonstrates high selectivity and reproducibility, with stable system operation.

However, at 300 rpm, sorting performance declines. Excessive centrifugal force causes significant deviation in droplet trajectories, and small droplets may fail to enter the slit in time, resulting in sorting errors. Additionally, flow field stability is disturbed, reducing the chip’s repeatability and robustness.

In summary, rotation speed significantly affects droplet migration efficiency and sorting limit by modulating inertial behavior and flow paths within the chip. Under the experimental conditions, 200 rpm is identified as the critical parameter for achieving optimal sorting performance, providing valuable guidance for microfluidic chip design and operation.

### 3.6. Cross-Contamination Verification

To further validate the chip’s capability in sorting complex droplet samples, we conducted experiments using droplets encapsulating microparticles. This setup simulates typical droplet structures found in practical applications, such as cells, microspheres, or particle suspensions in heterogeneous systems. Specifically, we used 10 μm polystyrene microspheres at a concentration of 2.5% (in 10 mL volume) to prepare oil-in-water droplets via mechanical agitation. The sorting process was then carried out using the microfiltration chip configured with a ramp angle of 30° and a gap depth of 200 μm, under a centrifugal rotation speed of 100 rpm.

**Figure 14:**
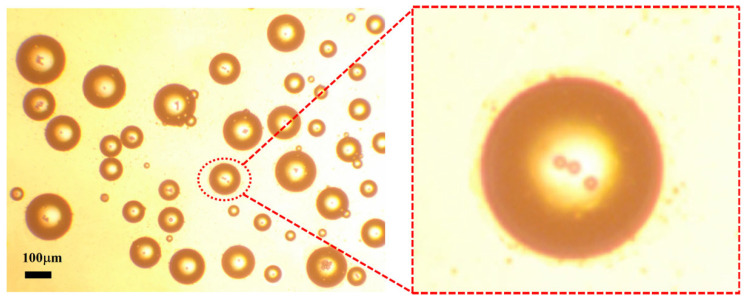
Performance of particle-encapsulated droplet sorting using oil-in-water emulsion structures.

As shown in Figure 14, the chip successfully recognized and selectively sorted droplets based on their encapsulation status. Encapsulated droplets exhibited significant lateral deviation under the combined effect of centrifugal force and structural guidance, allowing them to be effectively separated from unencapsulated ones. This result further demonstrates the chip’s adaptability and reliability in processing structurally heterogeneous droplet systems and supports its potential in applications such as cell encapsulation and microreactor construction.

**Figure 15:**
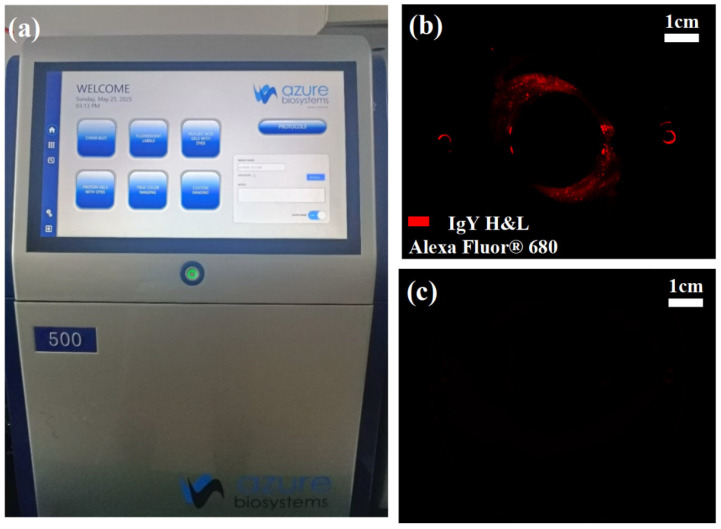
(**a**) Azure 500 bioimaging system. (**b**) Fluorescence signal of cross-contamination detection without elution. (**c**) Fluorescence signal of cross-contamination detection with elution.

To evaluate the chip’s ability to control cross-contamination during sorting, this study employed the Azure 500 bioimaging system (manufactured by Azure Biosystems, Dublin, CA, USA) to analyze the distribution and intensity of fluorescence signals within the chip channels, as shown in Figure 15a. Alexa Fluor 680-labeled IgY H&L antibodies (at a concentration of 1 μg/mL) were encapsulated within oil-in-water microdroplets to simulate target samples with a high fluorescence background. These droplets were then injected into the rotary microfilter chip and operated at the standard sorting speed of 100 rpm.

To assess the fluorescence residue inside the chip after sorting, two experimental conditions were set for comparison: the non-elution group, in which fluorescence imaging was performed immediately after sorting without any additional treatment; and the elution group, where the chip was subjected to high-speed rotation at 600 rpm for 30 s post-sorting to enhance the continuous phase fluid’s flushing effect via centrifugal force before fluorescence imaging.

As shown in Figure 15b, noticeable fluorescence residue remained in the curved channel and slit entrance regions of the non-elution group, indicating significant sample retention in structural dead zones and posing a potential risk of cross-contamination. In contrast, Figure 15c demonstrates that after high-speed elution, fluorescence signals within the chip channels were nearly eliminated, confirming the effectiveness of the elution process in removing residual droplets and effectively suppressing cross-contamination.

**Figure 16:**
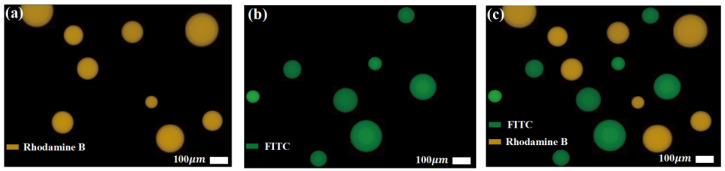
Verification of cross-contamination between dual fluorescently labeled samples: (**a**) fluorescence image under Rhodamine B excitation in the mixed sample; (**b**) fluorescence image under FITC excitation; (**c**) merged image of both fluorescent channels. The distinct separation of the two fluorescent signals indicates minimal cross-contamination within the chip.

To further verify sorting purity, two fluorescently labeled droplet samples were prepared: 1 mg/mL Rhodamine B and 0.1 mg/mL FITC, as shown in Figure 16a,b, respectively. These samples were sequentially introduced into the same chip and subjected to dual-sample sorting. A centrifugal rinsing step was performed after each sorting round to remove residual droplets. Considering Rhodamine B’s tendency to precipitate in mineral oil, silicone oil was used as the continuous phase to maintain system stability. The collected droplets were diluted, transferred to glass slides, and imaged using an inverted fluorescence microscope (Leica DMi8, Leica Microsystems, Wetzlar, Germany).

As shown in Figure 16c, no overlap or interference between the two fluorescence signals was observed, indicating that no cross-contamination occurred during the dual-sample sorting process. This is particularly significant for biological applications, where samples often exist as heterogeneous emulsions. The demonstrated sorting strategy offers a feasible solution for high-efficiency, contamination-free isolation of heterogeneous droplet microreactor units and holds strong promise for practical implementation.

In summary, the synergistic integration of the composite structural design and the centrifugal rinsing mechanism significantly enhances the sorting purity and biosafety of the chip during high-throughput operations. The system demonstrates strong capability for multi-sample processing and broad applicability across diverse scenarios. This strategy holds great promise for advancing microdroplet analysis and the handling of complex biological samples, facilitating the practical deployment of microfluidic systems in medical diagnostics, drug screening, and environmental monitoring. In the future, it may serve as a critical component within standardized operational workflows for microfluidic platforms.

## 4. Conclusions

This study proposes and experimentally validates a centrifugal-force-driven weir-type crossflow microfilter chip, which achieves efficient and highly selective sorting of droplets with varying diameters through the synergistic design of a wedge-shaped slope and slit structure. Experimental results demonstrate that the system exploits the size-dependent response of droplets in the centrifugal field to selectively allow droplets below a critical diameter to pass while effectively excluding larger droplets, thereby realizing size-based separation. Mechanistic analysis reveals that droplets smaller than the critical threshold can smoothly traverse the slit, whereas larger droplets deviate from the main channel due to the coupling of centrifugal force and slope-induced inertial displacement, leading to their effective ejection.

Following sorting, a brief centrifugal elution step is implemented by injecting buffer solution and temporarily increasing the rotational speed, which efficiently removes residual droplets within the channels, significantly alleviates microchannel clogging, and enhances system repeatability and biosafety.

The chip has been successfully applied to the screening of deformable droplet systems. Results indicate a unimodal droplet size distribution, with larger slope angles (φ) and slit depths (h) favoring increased sorting purity, while relatively smaller structural parameters enhance sorting precision. Balancing sorting efficiency and accuracy, the optimal structural parameters were identified as φ = 30° and h = 200 μm. Building upon this, the influence of rotational speed (ω) was further investigated. Experiments show that at 200 rpm, the system reaches optimal performance, achieving a sorting rate of approximately 72% and a sorting limit near 98%. The study also reveals a significant coupling relationship among the critical sorting diameter (DDC), slope angle φ, slit depth h, and rotational speed ω, providing experimental guidance for multi-parameter co-optimization.

Additionally, a mechanical equilibrium model of droplets on the slope was developed to derive the critical condition for inertial displacement, offering theoretical support for the observed sorting behavior. Finally, fluorescence residue imaging experiments verified the chip’s robust anti-contamination capability when handling samples with high fluorescence background, further reinforcing its practicality and safety in high-throughput multi-sample processing.

In summary, the proposed centrifugal microfilter chip demonstrates excellent scalability and application potential in microfluidic scenarios such as high-throughput droplet screening, biological sample preprocessing, and single-cell manipulation. It holds promise for broader engineering applications and standardized deployment in medical diagnostics, drug screening, and environmental analysis.

## Figures and Tables

**Table 1:** Physical Properties of the Liquid Phase Used in Experiments.

Liquid	(*ρ*, kg/m^3^)	(*μ*, mPa·s)	(*σ*, mN/m)
Water	1000	0.89	71.7
#5 mineral oil	823.4	18.11	22.0

## Data Availability

Data will be made available on request.
